# Evaluation d’une grille de supervision des laboratoires des leishmanioses cutanées au Maroc

**DOI:** 10.11604/pamj.2017.27.253.11385

**Published:** 2017-08-04

**Authors:** Bouchra El Mansouri, Fatima Amarir, Yamina Hajli, Hajiba Fellah, Faiza Sebti, Bouchra Delouane, Abderrahim Sadak, El Bachir Adlaoui, Mohammed Rhajaoui

**Affiliations:** 1 Département de Parasitologie, Institut National d’Hygiène, Agdal, Rabat, Maroc; 2 Université Mohamed V, Faculté des Sciences de Rabat, Maroc

**Keywords:** Evaluation, liste de contrôle, supervision de laboratoire, leishmanioses, Maroc, Assessment, control list, supervision of the laboratory, leishmaniasis, Morocco

## Abstract

**Introduction:**

Afin d’évaluer une grille de contrôle standardisée de laboratoire de diagnostic des leishmanioses, comme nouveau outil de supervision.

**Méthodes:**

Un essai pilote a été pratiqué sur sept laboratoires provinciaux, appartenant à quatre provinces au Maroc, en suivant l’évolution de leurs performances tous les deux ans, entre l’année 2006 et 2014. Cette étude détaille la situation des laboratoires provinciaux avant et après la mise en œuvre de la grille de supervision. Au total vingt et une grille sont analysées.

**Résultats:**

En 2006, les résultats ont montré clairement une insuffisance des performances des laboratoires: besoin en formation (41.6%), personnel pratiquant le prélèvement cutané (25%), pénurie en matériels et réactifs (65%), gestions documentaire et local non conformes (85%). Différentes actions correctives ont été menées par le Laboratoire National de Référence des Leishmanioses (LNRL) durant la période d’étude. En 2014, le LNRL a enregistré une nette amélioration des performances des laboratoires. Les besoins en matière de formation, qualité du prélèvement, dotation en matériels et réactifs ont été comblés et une coordination efficace s’est établie entre le LNRL et les laboratoires provinciaux.

**Conclusion:**

Ceci montre l'efficacité de la grille comme outil de supervision de grande qualité, et comme pierre angulaire de tout progrès qui doit être obtenu dans les programmes de lutte contre les leishmanioses.

## Abstract (en)

**Introduction:**

The aim of our study was to assess a standardized supervisory grid as a new supervision tool being used in the laboratories of leishmaniasis.

**Methods:**

We conducted a pilot trial to evaluate the ongoing performances of seven provincial laboratories, in four provinces in Morocco, over a period of two years, between 2006 and 2014. This study detailed the situation in provincial laboratories before and after the implementation of the supervisory grid. A total of twenty-one grids were analyzed.

**Results:**

In 2006, the results clearly showed a poor performance of laboratories: need for training (41.6%), staff performing skin biopsy (25%), shortage of materials and reagents (65%), non-compliant document and local management (85%). Several corrective actions were conducted by the National Reference Laboratory (LNRL) of Leishmaniasis during the study period. In 2014, the LNRL recorded a net improvement of the performances of the laboratories. The need for training, the quality of the biopsy, the supply of tools and reagents were met and an effective coordination activity was established between the LNRL and the provincial laboratories.

**Conclusion:**

This trial shows the effectiveness of the grid as a high quality supervisory tool and as a cornerstone of making progress on fight programs against leishmaniases.

## Introduction

Les leishmanioses sont des maladies infectieuses transmises par des diptères piqueurs, les phlébotomes. Elles sont déterminées par un groupe complexe de protozoaires *kinetoplastida* du genre *Leishmania*. On connaît actuellement environs 17 espèces de leishmanies réparties dans 88 pays et quatre continents [1]. Les leishmanioses sont des parasitoses endémiques du Maroc. Elles étaient découvertes depuis le début du 20^ème^siècle. Les études éco-biologiques antérieures des leishmanies ont révélé la présence simultanée de 3 types de leishmanioses dans le royaume, à savoir: (a) La Leishmaniose Viscérale (LV) due à *L. infantum* est répartie principalement au Nord du Maroc, (b) La Leishmaniose Cutanée (LC) due à *L. major*, régnant dans le Sud et le Sud-Est de la chaîne de l'Atlas marocain, et enfin, la LC à *L. tropica* occupant jusqu’à maintenant le centre et le Maroc méridional [2].

Le Ministère de la Santé a instauré un programme national de lutte contre les leishmanioses (PNRL) en 1996. La lutte est basée sur le dépistage des cas, le traitement et la lutte contre les vecteurs et les réservoirs animaux (rongeurs et chiens). Le diagnostic repose sur l’examen microscopique des frottis cutanés [3]. Toutes les lames examinées à l’échelle nationale sont acheminées vers le LNRL pour contrôle et confirmation du diagnostic. Les animateurs des laboratoires assurent la gestion des laboratoires, mais ils manquent une grille de supervision standardisée des laboratoires des leishmanioses, en outre certains d'entre eux ne possédaient pas encore les connaissances suffisantes en techniques de prélèvements, diagnostic, qualité, hygiène et sécurité au laboratoire, pour pouvoir assurer une supervision efficace. Dans l’objectif d’étudier et d’améliorer les performances des laboratoires du réseau national, nous avons élaborée une liste standardisée de contrôle des laboratoires des leishmanioses cutanées et nous avons pratiqué un essai-pilote de la grille sur quatre provinces, abritant sept laboratoires, de l’année 2006 à 2014. L’étude détaille la situation des laboratoires avant et après la mise en œuvre de la liste de contrôle standardisée.

## Méthodes

### Réseaux des laboratoires

Le réseau des laboratoires comporte un Laboratoire National de Référence des Leishmanioses (LNRL), crée en 1997 à l’Institut National d’Hygiène de Rabat, et un ensemble de 61 laboratoires périphériques des leishmanioses rattachées aux services ambulatoires provinciaux, aux hôpitaux ou aux centres périphériques de santé dans les cinquante neuf provinces marocaines. Le choix des laboratoires de l’étude était aléatoire représentant les quatre pôles géographiques du Maroc : Essaouira du Sud, Settat et Béni Mellal du centre et Tétouan du Nord.

### Liste de contrôle

Nous avons mis en place la liste de contrôle pour la supervision du laboratoire qui a été discutée et approuvée par les membres du LNRL. Les superviseurs ont été formés à compléter correctement la liste de contrôle. L’hors des missions de supervision, le superviseur a eu à sa disposition une voiture de service avec un chauffeur. Les dépenses de carburant, de réparation et frais de déplacements ont été couvertes par le PNLL. La liste de contrôle comporte deux sections: (a) La section générale envisage les problèmes suivants : local, besoins généraux du laboratoire en réactifs, matériel et équipement, mesures de sécurité, évacuation des déchets, formulaires de demande de laboratoire et registres, envoi des lames pour le contrôle de qualité, besoins de formation et charge de travail. (b) La section technique, couvre les aspects techniques de prélèvement, préparation de frottis, l’examen microscopique et le contrôle de qualité externe. La dernière partie soulève les recommandations et actions correctives opérationnelles réalisées par chaque superviseur.

### Période d’étude

La mise en route (ou phase -pilote) de la liste de contrôle des supervisions s'est étalée sur trois phases : 2006-2008, 2011-2012 et 2013-2014. Les superviseurs ont rempli 21 listes de contrôle à partir de 7 laboratoires dans quatre provinces.

### Traitement des données

Les listes de contrôle ont été enregistrées dans une base de données spécialement élaborée avec le logiciel Excel où les performances du laboratoire ont été suivies. L'objectif à atteindre, en vue d'une amélioration certaine, a été défini comme le comblement des insuffisances à la fin de l’année 2014.

## Résultats

Nous avons analysé un total de 21 listes de contrôle. Elles représentaient les observations en provenance de 7 laboratoires dans quatre provinces au cours de la période d’étude de l’année 2006 à 2014:

### Dotation en matériels et réactifs

En 2006, on a relevé une pénurie d’un réactif de laboratoire ou d'autres fournitures dans tous les laboratoires. Au bout de l’année 2014, tous les laboratoires étaient dotés en matériels et réactifs manquants.

### Hygiène et sécurité au laboratoire

En 2006, 6 laboratoires, soit 86%, avaient un local non-conforme aux normes (manque de tabourets, des bureaux et/ou de réserve, de paillasses de coloration, de lecture,…). En 2014, suite aux recommandations des superviseurs aux délégués, 5 laboratoires non conformes se sont améliorés : Le personnel de tous les laboratoires utilise les gants, les blouses et les désinfectants. Néanmoins, durant la période 2006-2014, la plupart des laboratoires n'ont pas mis en place un système de gestion des déchets qui répond à la norme de sécurité et qualité, malgré les recommandations des superviseurs.

### Gestion documentaire

Tous les laboratoires provinciaux n’affichaient pas les modes opératoires du prélèvement, de coloration et de diagnostic. D’après les lots des lames de LC des provinces reçues au niveau du LNRL, seulement 2 provinces (40%) parmi les 5 provinces, utilisent les formulaires de demande de renseignements des patients et envoient les lames pour contrôle et confirmation au LNRL. Dès lors le laboratoire ne recevait pas les informations complètes concernant le patient. Par conséquent, le registre du laboratoire provincial ne pouvait pas être complété correctement. Ceci peut entraîner des erreurs des notifications des cas qui peuvent influencer les statistiques lors des études des leishmanioses. En 2014, le non affichage des modes opératoires (MO) était signalé dans 2 laboratoires parmi les 7.

### Prélèvements

Trois microscopistes parmi **12** interrogés (25%) ont effectué eux mêmes les prélèvements cutanés car tous les prélèvements reçus des centres périphériques, étaient de mauvaise qualité. Durant l'année 2014, les microscopistes des laboratoires ont fait les prélèvements cutanés eux mêmes et le personnel des C/S a profité des formations pratiques en matière des prélèvements dans 2 provinces.

### Techniques parasitologiques et formation

#### Préparation des frottis cutanés et lecture des lames

Le personnel de deux laboratoires a pratiqué le diagnostic des leishmanioses en appliquant la méthode de la préparation des frottis du paludisme, à cause de la mutation ou du départ à la retraite du personnel qualifié. Le prélèvement est généralement sanglant, fixé et coloré par Giemsa (3%) pendant 30 minutes, au lieu de 10% pendant 45 minutes. Cependant, le prélèvement doit contenir la sérosité riche en macrophages qui hébergent naturellement les leishmanies. Ces microscopistes ne lient pas tous les champs de la lame, mais se limitent à 100 champs suivant les consignes du diagnostic du paludisme. Ceci peut affecter la qualité du diagnostic vu que le frottis doit être bien étalé et bien coloré par Giemsa pour révéler la présence des amastigotes. D’autre part, un laboratoire ne faisait pas la fixation des lames par le méthanol par manque de réactif. Ces erreurs ont des conséquences négatives sur la conservation des lames jusqu’à l'arrivée à l’INH pour confirmation et contrôle. Au total 5 microscopistes ont été signalés ayant un besoin urgent en formation (41%).

### Envoi des lames de LC au LNRL

En général, la plupart des techniciens de laboratoire n'étaient pas informés de l'importance de l'envoi du 100% des lames au LNRL pour contrôle et confirmation. En 2014, tous les laboratoires ont envoyé presque la totalité des lames.

### Application de la liste de contrôle

Le tableau détaille les insuffisances identifiées dans sept laboratoires au cours de l’année 2006 avant l'utilisation de la liste de contrôle et les observations de ces secteurs obtenues après 7 ans d'utilisation systématique de la liste de contrôle par les superviseurs du LNRL. Ces données montrent qu'après une utilisation systématique de la liste de contrôle, presque tous les laboratoires se sont améliorés par comparaison à la situation initiale (Tableau 1).

**Tableau 1:** Améliorations suite à l'utilisation des listes de contrôle par les laboratoires périphériques dans 4 provinces du Maroc de 2006 à 2014

Paramètres		AVANT	APRES	
		2006-2008	2011-2012	2013-2014
**Local non conforme**	Nombre de laboratoires manquant de réserve, tabouret ou paillasse de coloration	71% (5/7)		14.4 (1/7)
	Nombre de laboratoires manquant de paillasse de lecture	14.4% (1/7)		0
**Manque matériels, réactifs et entretien**	Pénurie en réactifs	65%	5%	
	Manque d’appareil à eau distillée/microscope	57% 4/7		28% 2/7
**Manque de MO affiches**		100%		28% 2/7
**Manque de mesure de sécurité**	Blouses	14.4%	0%	0%
	Désinfectant	0%	0%	0%
	Tri des déchets	100%	100%	100%
**Prélèvement**	Microscopiste	25%	25%	100%
	Médecin	50%	25%	25%
	Infirmiers	25%	10%	10%
	C/S externes formés en prélèvements	0%	40%	14.4%
**Besoin en formation**		41.6% 5/12	0%	8.3%

## Discussion

Au Maroc, la situation des laboratoires provinciaux des leishmanioses en 2006, a montré clairement une insuffisance des performances traduites par le manque d’une ou plusieurs fournitures ou réactifs de laboratoire. Ceci a permis de dévoiler la situation de certaines provinces ayant plusieurs laboratoires périphériques et qui ne font pas une distribution équitable de la fourniture. Un des laboratoires périphériques supervisés manque toujours d’appareil à eau distillée, microscope, et tabouret. Pourtant, le microscopiste, ayant suivi une formation locale en matière des leishmanioses, a une charge de travail importante, et utilise le microscope doté par le programme de tuberculose. Ceci engendre de sérieuses inquiétudes sur le fonctionnement des laboratoires périphériques sectorielles. L’action corrective a permis de spécifier la destination des dotations en matériels et réactifs à l’échelle sectorielle suivant les recommandations des superviseurs.

Le manque du personnel non remplacé à cause des mutations et retraites, le manque de formation de certains infirmiers font que les prélèvements cutanés des patients sont généralement délégués aux hôpitaux ou aux infirmiers des centres de santé qui le font occasionnellement selon leurs connaissances ultérieures non-conformes avec les directives du LNRL. Par conséquent, les patients doivent parcourir de longues distances pour faire l’analyse et le traitement de leurs lésions. Parfois le patient abandonne le suivi médical ce qui contribue à maintenir la transmission surtout dans les foyers à *L. tropica*.

A titre correctif, les superviseurs ont recommandé aux microscopistes et/ou aux dermatologues des quatre laboratoires provinciaux, de faire une formation pratique aux personnels des centres de santé. Une province a pris le relai et a doté le microscopiste d’une voiture de service afin de visiter les centres de santé, et former le personnel de laboratoire en matière de prélèvement, étalement ou confection des frottis et recensements des renseignements complets des patients sur les fiches. La formation a concerné 2 C/S et a pris une heure par C/S. La deuxième province a demandé un budget pour faire la formation aux C/S et les autres provinces sont restées indifférentes. Pour remédier à la situation, le LNRL a organisé en 2008 une formation aux profits des C/S d’une seule province vu le manque de budget.

Pour le personnel déjà formé à l’INH, les erreurs relevées au cours du processus d’étalement, de coloration et de lecture des lames ont suscité une stratégie corrective basée sur le recyclage des microscopistes et une augmentation de la durée des séances de travaux pratiques lors des sessions de formation organisées par le LNRL. Aussi, la circulaire de la DELM a été envoyée aux différents laboratoires afin que toutes les lames positives et négatives diagnostiquées avec des fiches de renseignements complètes soient adressées pour contrôle, confirmation et identification des souches dans le but d’actualiser les données sur les espèces circulantes des secteurs les plus touchés et ainsi bien orienter la stratégie de lutte.

Le LNRL a organisé un planning de formation au profit du personnel des laboratoires provinciaux, en 2006, 2009 et 2010 ce qui a diminué l’effectif des personnes non formés et a amélioré l’activité des laboratoires. Néanmoins, certaines personnes formées ont été mutées ou ont pris leur retraite entre 2010 et 2012, il restait donc 8.3% du personnel qui demande une formation continue ou de base. Tous les laboratoires sont dotés en documents nécessaires telles les fiches de renseignements, les modes opératoires et les fiches de contrôle de qualité et d’organisation des laboratoires. Les données obtenues des supervisions dans les sept laboratoires où a été réalisée pendant 7 ans, 3 supervisions successives (2006-2008, 2011-2012, 2013-2014), utilisant la liste de contrôle exhaustive, ont montré une nette amélioration des performances des laboratoires et microscopistes. Ceci peut être considéré comme la preuve de l'engagement du personnel de santé au Maroc et de leur détermination à améliorer leurs performances une fois en possession de moyens suffisants et d'une formation solide et continue. Ceci met également au premier plan l'importance d’un réseau de supervision décentralisée, qui pourra diminuer le coût des missions de supervision du LNRL, garantir le bon fonctionnement continu des laboratoires provinciaux et l’amélioration de la performance du personnel de santé en vue de produire des services de qualité.

## Conclusion

L'utilisation d'une liste de contrôle standardisée pour la supervision des laboratoires périphériques a montré un impact positif et a conduit a un progrès important vers une amélioration de la performances des laboratoires périphériques du Maroc, grâce à la correction sur place ou avec le temps de toute insuffisance et défaillance identifiées en matière de formation, de dépistage des cas, des matériels et/ou des réactifs. Cette liste de contrôle est un outil standardisé utile pour la supervision régulière des laboratoires et pour la surveillance des performances des centres de santé de diagnostic.

### Etat des connaissances actuelles sur le sujet

- Les supervisions des laboratoires des leishmanioses est une recommandation de l'OMS pour maintenir la qualité de diagnostic des leishmanioses cutanées et viscérales au Maroc;
- La grille de supervision est une base primordiale pour l'amélioration continue des programmes de lutte contre les leishmanioses.

### Contribution de notre étude à la connaissance

- Standardisation et évaluation d'une grille de supervision sensible et spécifique aux laboratoires des leishmanioses au Maroc;
- Etude de l'impact de cette grille sur la qualité du programme nationale de lutte contre les leishmanioses au Maroc;
- Mise en route de plusieurs indices d'évaluation interne du programme nationale de lutte contre les leishmanioses au Maroc qui peut servir de modèle a tout les pays.

## Conflits d’intérêts

Les auteurs ne déclarent aucun conflit d'interêt.
